# Twisting vortex lines regularize Navier-Stokes turbulence

**DOI:** 10.1126/sciadv.ado1969

**Published:** 2024-09-13

**Authors:** Dhawal Buaria, John M. Lawson, Michael Wilczek

**Affiliations:** ^1^ Department of Mechanical Engineering, Texas Tech University, Lubbock, TX 79409, USA.; ^2^ Max Planck Institute for Dynamics and Self-Organization, 37077 Göttingen, Germany.; ^3^ Department of Aeronautics and Astronautics, University of Southampton, Southampton SO17 1BJ, UK.; ^4^ Theoretical Physics I, University of Bayreuth, 95440 Bayreuth, Germany.

## Abstract

Fluid flows are intrinsically characterized via the topology and dynamics of underlying vortex lines. Turbulence in common fluids like water and air, mathematically described by the incompressible Navier-Stokes equations (INSE), engenders spontaneous self-stretching and twisting of vortex lines, generating a complex hierarchy of structures. While the INSE are routinely used to describe turbulence, their regularity remains unproven; the implicit assumption being that the self-stretching is ultimately regularized by viscosity, preventing any singularities. Here, we uncover an inviscid regularizing mechanism stemming from self-stretching itself, by analyzing the flow topology as perceived by an observer aligned with the vorticity vector undergoing amplification. While, initially, vorticity amplification occurs via increasing twisting of vortex lines, a regularizing anti-twist spontaneously emerges to prevent unbounded growth. By isolating a vortex, we additionally demonstrate the genericity of this self-regularizing anti-twist. Our work, directly linking dynamics of vortices to turbulence statistics, reveals how the Navier-Stokes dynamics avoids the development of singularities even without the aid of viscosity.

## Teaser

Analysis of how vortex lines twist in turbulent flows identifies the physical mechanism underpinning Navier-Stokes regularity.

## INTRODUCTION

Vortices, constituting regions of pronounced rotational coherence, are intrinsic to all fluid flows, both classical and quantum, and various other physical phenomena involving plasmas and electromagnetism (*1*–*6*). From collisions of microscopic water droplets in clouds (*7*) to global circulation patterns on planets and stars (*8*) and from nanodevices (*9*) to aerospace vehicles (*10*), they play a critical role in all natural and man-made hydrodynamic phenomena. Mathematically, vortices are characterized by the vorticity vector **ω** = ∇ × **u**, where **u** is the velocity field; thus, fluid flows can be inherently described as an ensemble of vortex lines, akin to streamlines depicting the velocity field. Their evolution is routinely described by the incompressible Navier-Stokes equations (INSE), written in the vorticity form

$$\frac{D\omega }{\mathrm{Dt}}=\omega \cdot \nabla u+\nu \nabla^{2}\omega$$ (1)

where *D*/*Dt* = ∂*_t_* + **u** · ∇ is the material derivative, ν is the kinematic viscosity, and incompressibility implies ∇ · **u** = 0, i.e., velocity is solenoidal.

**Fig. 1.**
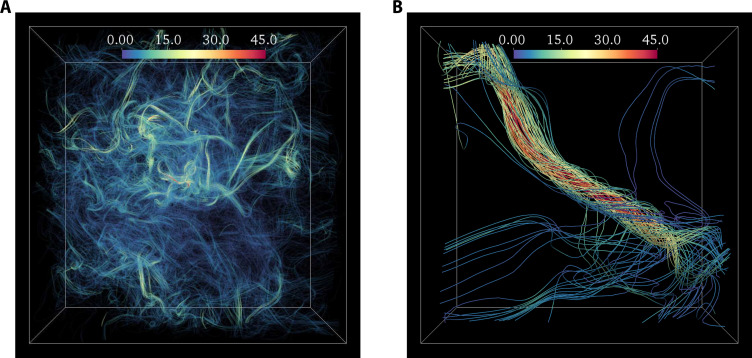
Vortex lines in turbulence. (**A**) The structure of vortex lines in instantaneous vorticity field from DNS of turbulence at *R*_λ_ = 1300. The size of the domain shown is (256η)^3^, where η is the Kolmogorov length scale. The vortex lines are color coded with vorticity magnitude (as normalized by the rms). The visualization reveals bundling of vortex lines around intense events, into well-known tube-like structures. (**B**) A zoomed-in view around the center of domain in (A) shows an individual vortex bundle around the intense event demonstrating conspicuous twisting of vortex lines. The size of the subdomain in (B) is (50η)^3^.

Describing a wide range of fluid dynamical phenomena, the INSE are of central importance in science and engineering and are routinely used in a wide array of numerical simulations (*11*). However, from a mathematical standpoint, it is still unknown whether they are well posed, i.e., whether solutions to INSE always remain smooth or develop singularities in finite time, which would preclude their widespread usability. Consequently, their regularity problem has been recognized by the Clay Mathematics Institute as one of the Millennium Prize problems (*12*, *13*). The possibility of a finite-time singularity arises from the nonlinear term **ω** · ∇ **u** in Eq. 1, which prescribes amplification of vorticity via self-stretching of vortex lines (or vortex stretching), whereas the viscous term acts to oppose this amplification (*14*–*16*). It follows that for a singularity, the nonlinear term must grow unbounded (*17*) which, if possible, can only happen when the viscosity is sufficiently small (*13*). This corresponds to the turbulent regime, a far-from-equilibrium state characterized by chaotic multiscale fluctuations, leading to intermittent generation of extreme vorticity events and a highly complex spatiotemporal structure of underlying vortex lines (*1*, *18*–*20*). Figure 1 illustrates the structure of vortex lines in turbulence, revealing prevalence of a hierarchy of coherent vortex structures. In regions of intense vorticity, these structures consist of bundles of high-amplitude vortex lines, which display an inner twist, among other topological properties.

The regularity problem for the INSE and intermittency in turbulent flows are intimately connected (*13*, *21*–*23*). However, both problems remain inherently challenging due to underlying mathematical difficulties in deciphering the nonlinear amplification term in Eq. 1. It is worth noting that this term is closed in vorticity, since velocity can be obtained from vorticity via the Biot-Savart integral over the flow domain

$$u\left(x,t\right)=\frac{1}{4\pi }\int_{r}\frac{r}{{|r|}^{3}}\times \omega \left(x+r,t\right)\;dr$$ (2)

This nonlocal integral thus not only couples all the scales in the flow but also renders Eq. 1 analytically intractable, posing a serious challenge in addressing the nonlinearity (*13*). Some understanding of this nonlocality can be obtained by evaluating the Biot-Savart integral from numerical simulations of INSE and analyzing its behavior in the neighborhood of intense vorticity, see, e.g., (*16*, *24*, *25*). From a geometrical standpoint, a singularity is engendered by untamed stretching and twisting of vortex lines, ultimately rendering them nonsmooth (*26*, *27*) and simultaneously leading to unbounded growth of the nonlinear term. While recent works (*23*, *25*) have argued against unbounded growth, they do not provide the necessary geometrical and structural understanding of the flow around intense vorticity or any dynamical information on evolution of vortex lines as a whole—which is essential to understand the behavior of the nonlinearity in the INSE. Such a task is inherently challenging due to the complex hierarchy of vortex structures prevalent in the turbulent regime and often instead restricted to simplified vortex flows with specific initial conditions (*28*, *29*).

In this work, by taking the perspective of an observer aligned with vorticity undergoing amplification, we isolate the topology and dynamics of vortex lines around it to highlight the physical mechanism underpinning the regularity of Navier-Stokes turbulence. In such a conditional reference frame, vorticity amplification necessitates that vortex lines have a degree of twist around the observer (*30*, *31*). However, we demonstrate that as vorticity is increasingly amplified, an anti-twist is spontaneously generated within the vortex core which attenuates further amplification. The robustness of this observation is further verified by isolating an individual vortex, which is then evolved using the INSE, once for finite viscosity and once with viscosity explicitly set to zero (effectively simulating Euler equations). In both cases, the same qualitative behavior is obtained, with the temporal dynamics demonstrating the emergence of the self-regularizing anti-twist as vorticity is amplified. This mechanism provides a natural explanation for the recently identified self-attenuation of extreme vorticity events in turbulence (*23*) and elucidates how the nonlinear dynamics of Navier-Stokes equations preclude formation of any singularities.

## RESULTS

### CAV framework

The change in reference frame is accomplished by using the so-called conditionally averaged vorticity (CAV) field

$$\bar{\omega }\left(r,\zeta \right)=\langle \omega \left(x+r,t\right)|\zeta =\omega \left(x,t\right)\rangle$$ (3)

which essentially captures how an observer aligned with vorticity in a particular state **ζ** = **ω**(**x**, *t*) at any location **x** (and time *t*) and perceives the neighboring vorticity field **ω**(**x** + **r**, *t*) at any distance **r** from **x**. The average 〈·〉 is taken over multiple independent realizations (we elaborate further on this later). The CAV field was originally proposed by Novikov (*30*) and has been used in the context of turbulence theory and closure modeling (*30*–*33*). By aligning with the vorticity vector **ω**(**x**, *t*) undergoing amplification, the CAV field also allows us to analyze the flow topology in its neighborhood. To this end, we consider the CAV field in cylindrical polar coordinates (ρ, θ, *z*)

$$\bar{\omega }\left(r,\zeta \right)={\bar{\omega }}_{z}e_{z}+{\bar{\omega }}_{\rho }e_{\rho }+{\bar{\omega }}_{\theta }e_{\theta }$$ (4)

where the orthogonal unit vectors are given as $e_{z}=\hat{\omega }=\zeta /|\zeta |$ , $e_{\rho }=\left(\hat{r}-\gamma e_{z}\right)/{\left(1-\gamma^{2}\right)}^{1/2}$ , and **e**_θ_ = **e***_z_* × **e**_ρ_, with $\hat{r}=r/|r|$ and $\gamma =\hat{r}\cdot e_{z}$ . The vorticity condition **ζ** = **ω**(**x**, *t*) is always centered at the origin **r** = 0 in this frame and points along **e***_z_*. It simply follows that the coordinates ρ = **r** · **e**_ρ_ = *r*(1 − γ^2^)^1/2^, and *z* = **r** · **e***_z_* = *r*γ, with *r* = ∣**r**∣ = (ρ^2^ + *z*^2^)^1/2^. For convenience, we also define the enstrophy Ω = ∣**ω**(**x**)∣^2^, which simply quantifies the magnitude of the vorticity undergoing amplification.

In general, the CAV field is a function of vectors **r** and **ζ**, i.e., six variables; however, using statistical isotropy, the dependence is reduced to three scalar variables Ω, *r*, and γ (*31*), i.e., the magnitudes of **r** and **ζ** and the relative alignment between them. Thus, ${\bar{\omega }}_{z,\rho ,\theta }={\bar{\omega }}_{z,\rho ,\theta }\left(\Omega ,r,\gamma \right)$ or, alternatively, in the cylindrical polar coordinates: ${\bar{\omega }}_{z,\rho ,\theta }={\bar{\omega }}_{z,\rho ,\theta }\left(\Omega ,\rho ,z\right)$ , which implies that the CAV field is axisymmetric, naturally justifying the use of these coordinates.

Similar to the CAV field, one can also extract the conditional velocity field around **x** using the same procedure, i.e., 〈**u**(**x** + **r**, *t*)∣**ζ**〉. However, the velocity field can also be obtained from the CAV field using the Biot-Savart integral in Eq. 2. The nonlinear amplification term in Eq. 1 can then be obtained in terms of the CAV field (*30*, *31*). Since we are predominantly concerned with vorticity magnitude for a potential singularity, we first take the dot product of Eq. 1 with **ω**, which gives an equation for Ω. The nonlinear amplification term is then given as (see Materials and Methods for derivation)

$$\langle (\hat{\omega }\cdot \nabla u)\cdot \hat{\omega }|\Omega \rangle =\int_{0}^{\infty }\int_{0}^{\infty }\frac{3\rho^{2}z}{r^{5}}\;{\bar{\omega }}_{\theta }\left(\Omega ,\rho ,z\right)\;d\rho \;\mathrm{dz}$$ (5)

The nonlinear stretching of vorticity can be solely written in terms of ${\bar{\omega }}_{\theta }$ , which represents the twist associated with the CAV field. Note that this connection was already realized in earlier works (*30*, *31*), albeit formulated in spectral space; whereas, here, we have reformulated it in physical space (*32*), which is more intuitive and useful for the subsequent discussion. This expression is also linear in ${\bar{\omega }}_{\theta }$ , which provides remarkable simplification in analyzing the nonlinearity when using the CAV framework. The full nonlinear term can be obtained as $\langle \left(\omega \cdot \nabla u\right)\cdot \omega |\Omega \rangle =\Omega \langle \left(\hat{\omega }\cdot \nabla u\right)\cdot \hat{\omega }|\Omega \rangle$ . We have also used the fact that ${\bar{\omega }}_{\theta }\left(z\right)=-{\bar{\omega }}_{\theta }\left(-z\right)$ , i.e., ${\bar{\omega }}_{\theta }$ is an odd function of *z*, which follows from rotational symmetry of the CAV field.

From Eq. 5, it is evident that for the nonlinear stretching to be positive, the integral on the right-hand side must be positive, leading to the expectation that ${\bar{\omega }}_{\theta }\left(z\right)>0$ for *z* > 0 [and ${\bar{\omega }}_{\theta }\left(z\right)<0$ for *z* < 0 from symmetry]. Thus, we anticipate a positive twist of the CAV, which increases in strength as the nonlinear amplification increases to enable vorticity amplification. We will indeed demonstrate this result in the next section. However, we will demonstrate that when vorticity magnitude is sufficiently large, a negative anti-twist emerges, i.e., ${\bar{\omega }}_{\theta }\left(z\right)<0$ for some *z* > 0, in conjunction with positive twist in the background, which then attenuates further vorticity amplification. It is worth emphasizing that the twist component ${\bar{\omega }}_{\theta }$ explicitly relates to the nonlinear term only, i.e., it does not contribute to the viscous term (*31*). Thus, the emergence of anti-twist explicitly amounts to an inviscid regularizing mechanism originating from the nonlinearity itself.

### Data

To obtain the CAV field and associated quantities, we use data from both laboratory experiments and a large database generated via direct numerical simulations (DNS) of the INSE. The laboratory experiments correspond to turbulence measurements in a von-Kármán swirling water flow (*34*). The measurements are made in a small 1-cm^3^ volume near the mean-field stagnation point using scanning particle image velocimetry (PIV), allowing us to obtain the full three-dimensional (3D) velocity (and vorticity) field (*35*). The simulations correspond to the canonical setup of forced isotropic turbulence in a periodic domain and are performed using the well-known Fourier pseudo-spectral methods, allowing us to obtain any quantity of interest with highest accuracy practicable (*19*, *36*). The intensity of turbulence in both experiments and DNS is measured using the Taylor-scale Reynolds number *R*_λ_ ≡ *U*λ/ν, where *U* is the rms of velocity and λ is the Taylor length scale. Note that *R*_λ_ ∼ *Re*^1/2^ (*37*), where *Re* ≡ *UL*/ν is the large-scale Reynolds number, with *L* being the characteristic large scale. In experiments, *R*_λ_ ≈ 200, whereas in DNS, *R*_λ_ varied from 140 to 1300, on some of the largest grid sizes now feasible in turbulence simulations (*23*, *38*–*40*). In both cases, special attention is given to resolve the small scales and hence the intense vorticity events accurately (*20*, *38*), keeping the spatial resolution smaller than or equal to the Kolmogorov length scale η, which characterizes the cutoff scale at which viscosity regularizes the flow (*37*). Full details about both experiments and DNS are provided in Materials and Methods. The precise details about the extraction of the CAV field from the data are also provided there.

### Structure of CAV field

**Fig. 2.**
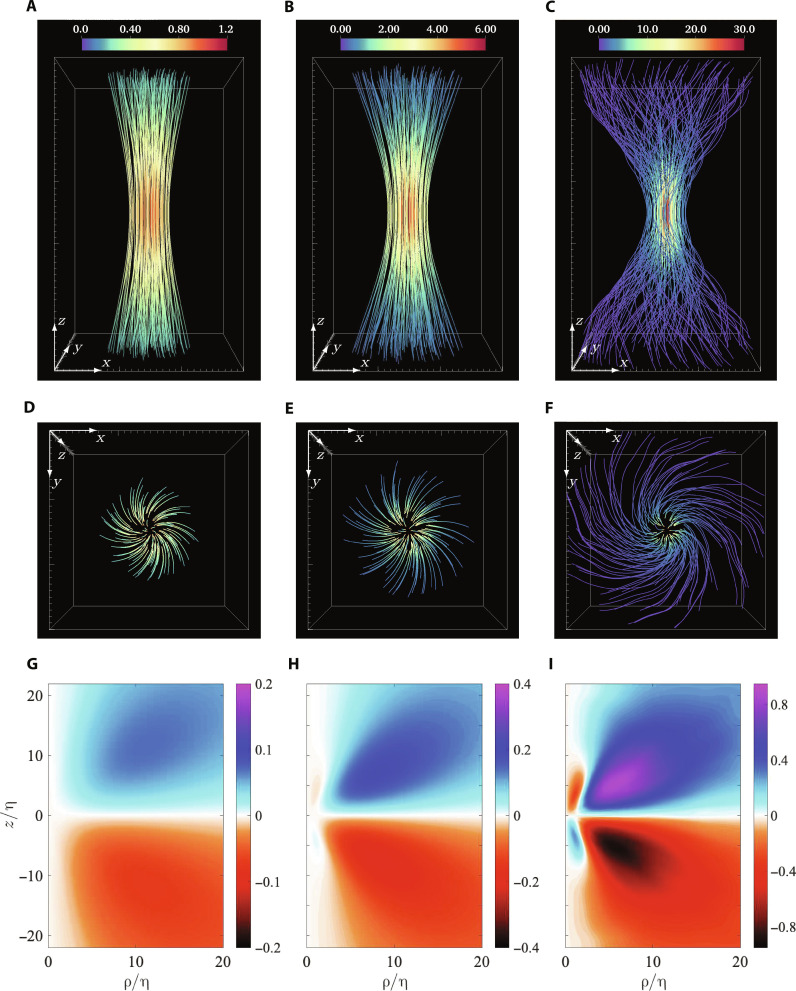
CAV field obtained from DNS. (**A** to **C**) Vortex lines showing the structure of the CAV field for increasing conditioning magnitudes of Ω/〈Ω〉 = 1, 30, 1000, respectively. The domain size in each case is 30η × 30η × 50η. Note that the condition vorticity **ζ** is at the center of the domain (corresponding to **r** = 0). (**D** to **F**) Top view of the domain in (A) to (C) highlighting the increasing twisting of vortex lines with the conditioning magnitude. (**G** to **I**) The twist component ${\bar{\omega }}_{\theta }$ of the CAV field, respectively, from axisymmetric planar cuts in (A) to (C). As the conditioning value increases, a clear anti-twist emerges in the center of the CAV field. (The components ${\bar{\omega }}_{z}$ and ${\bar{\omega }}_{\rho }$ are shown in fig. S1 for completeness).

Our first key result is shown in Fig. 2, which illustrates the structure of the CAV field with increasing magnitude of enstrophy. Fig. 2 (A to C) shows the visualization of the vortex lines in the neighborhood of the conditioned vorticity, which is always at the center of the domain and pointing upward (along the *z* axis). Each visualization reveals an elongated axisymmetric vortex with conspicuous twisting of vortex lines, which is concentrated near the center of the vortex and fans out along the elongated direction. The twisting also gets stronger and more prominent as the magnitude of the conditioned vorticity increases, which essentially captures the increased stretching required to generate more intense vorticity (*16*)—as also dictated by Eq. 5. To further highlight this aspect, Fig. 2 (D to F) shows the vortex structures in Fig. 2 (A to C), as viewed bottom to top (along the *z* axis). The increasing span of the vortex lines reiterates the increase in twist with the conditioning magnitude.

As noted earlier, the observation of a (positive) twist is expected on the grounds that vortex stretching is positive, and the observed structure of the CAV field is consistent with earlier results (*31*, *32*) in that regard. However, a notable previously overlooked feature is the anti-twist region that emerges for intermediate enstrophy condition and grows larger as enstrophy condition intensifies. While this aspect is difficult to observe from vortex lines, it is readily evident from the azimuthal component ${\bar{\omega }}_{\theta }$ of the CAV shown in Fig. 2 (G to I), corresponding to planar cuts through the mid-plane in both radial and axial directions from Fig. 2 (A to F). Evidently, the magnitude of ${\bar{\omega }}_{\theta }$ is overall stronger when the CAV field for stronger enstrophy is considered. Since net positive stretching is required to generate intense vorticity, it also follows from Eq. 5 that ${\bar{\omega }}_{\theta }$ is mostly positive for *z* > 0 [and also negative for *z* < 0 due to the odd symmetry ${\bar{\omega }}_{\theta }\left(\rho ,z\right)=-{\bar{\omega }}_{\theta }\left(\rho ,-z\right)$ ]. The negative anti-twist close to the CAV center opposes the positive background twist responsible for amplification of vorticity (and generation of enstrophy).

It is worth noting that the anti-twist observed in the CAV field also provides an explanation for the self-attenuation mechanism recently identified in (*23*). In this work, it was observed that in regions of very intense vorticity, the locally induced stretching is in fact negative and counteracts the net positive stretching. Our results here identify the anti-twist in the CAV as the key feature in the vorticity field that implies negative local stretching, essentially explaining the mechanism in (*23*).

Related to that, the twisted vortex structures identified in Fig. 2 (A to C) bear noticeable similarity to vortex tubes directly extracted from the instantaneous flow in Fig. 1. This is somewhat expected, since the CAV field essentially represents the typical local structures of the flow. In this context, it is worth mentioning that the vortex tubes in turbulence are often represented by axisymmetric Burgers vortices (*41*). However, Burgers vortices do not have any twisting and are incapable of self-amplification; instead, their enstrophy generation is facilitated via a constant background strain field. A similar observation was also made in (*23*), which identified local preferential alignment between velocity and vorticity, i.e., net positive helicity, in regions of intense vorticity. Once again, the twisting of vortex lines in the CAV field provides a natural explanation for this.

**Fig. 3.**
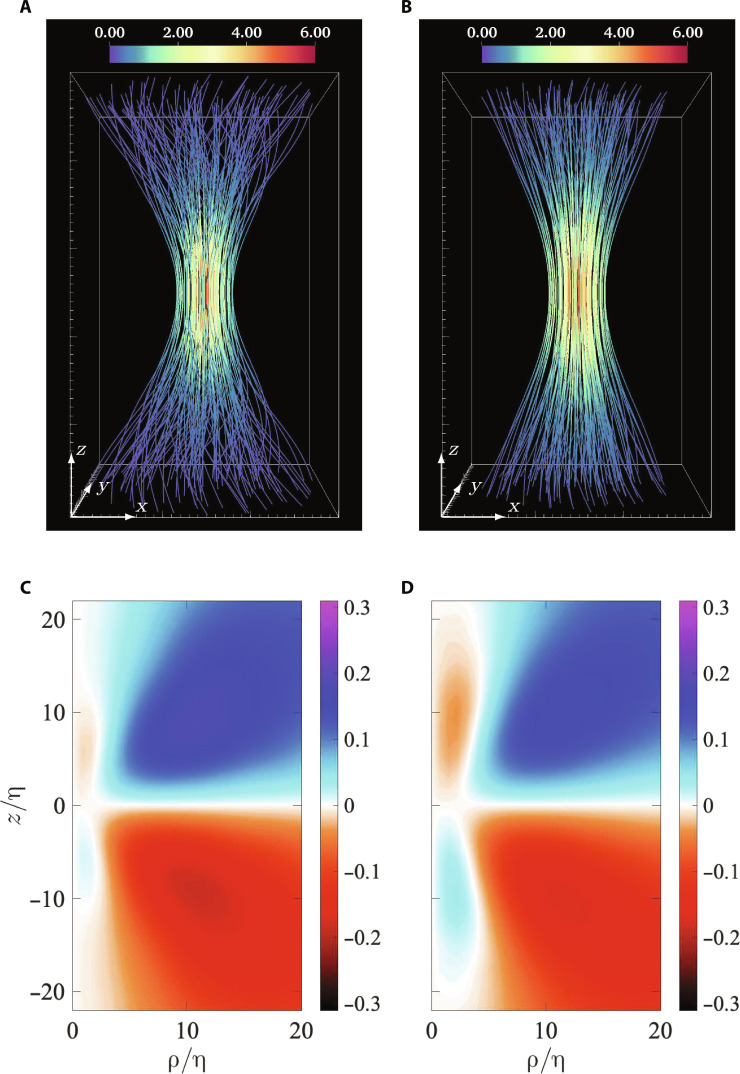
Comparing CAV fields from DNS and experiments. (**A** and **B**) Vortex lines showing the structure of the CAV field in DNS (A) and experiments (B). (**C** and **D**) The twist component ${\bar{\omega }}_{\theta }$ of the CAV field, respectively, from axisymmetric planar cuts in (A) and (B). The data in both cases correspond to *R*_λ_ = 200 and conditional Ω/〈Ω〉 = 30. (The components ${\bar{\omega }}_{z}$ and ${\bar{\omega }}_{\rho }$ are shown in fig. S2 for completeness, whereas fig. S3 presents a comparison at Ω/〈Ω〉 = 1, which does not have the anti-twist.)

To establish that this anti-twist is a generic feature of turbulence, we present a comparison of the DNS results to those obtained from a turbulent flow in laboratory experiments under very different conditions (see Materials and Methods for details). Figure 3 shows the vortex lines and the ${\bar{\omega }}_{\theta }$ corresponding to most intense vorticity available from DNS and experiments. The comparison is done at *R*_λ_ = 200, which is smaller than used in Fig. 2 (since higher *R*_λ_ are not available in our experiments). Nevertheless, as evident from Fig. 3, the development of the anti-twist at large conditional vorticity is an inherent property of turbulence. It is worth noting that the emergence of the anti-twist occurs at a lower enstrophy condition at lower Reynolds number. This is essentially because of intermittency, which dictates that the extremeness of an extreme enstrophy event (with respect to the mean value) increases with Reynolds number (*20*, *38*). A more detailed comparison between CAV fields from experiments and DNS is shown in the Supplementary Materials (in Figs. 2 and 3), comparing all the components of CAV, for two different vorticity conditions.

### Dynamics of an isolated vortex

The CAV results shown in Figs. 2 and 3 demonstrate the existence of an anti-twist region in the vortex core when vorticity is amplified to large magnitudes. However, they do not illustrate the dynamical origin of such an anti-twist. Since self-amplification of vorticity is an inherent characteristic of turbulence (*15*, *16*, *42*), it follows from Eq. 5 that the vortex lines develop a twist to enable this amplification. In the following, we demonstrate that the nonlinearity also inherently encodes the development of the countering anti-twist as a regularizing mechanism.

To this end, we perform a simple numerical experiment: We consider an isolated vortex structure extracted from the CAV field which, as an essential feature, only exhibits the regular twisting of vortex lines present (see Materials and Methods). This initial condition corresponds to a vortex structure with positive twisting everywhere, enabling self-amplification of vorticity. Essentially, we can write ${\bar{\omega }}_{\theta }>0$ for all *z* > 0 and ${\bar{\omega }}_{\theta }<0$ for all *z* < 0. Because of the axisymmetry of the CAV field, the vorticity vector undergoing maximum amplification is always at the center of the vortex core (**r** = 0) and pointed toward positive *z* axis. With this state as the initial condition, we then evolve it using the Navier-Stokes equations and also the inviscid Euler equations (*43*) (which corresponds to setting the viscosity to zero in the numerical simulation). In both cases, we demonstrate that an anti-twist naturally develops in the vortex core as vorticity is sufficiently amplified. It is worth noting that this approach is similar in essence to considering simplified vortex flows with arbitrary initial conditions, see e.g., (*28*, *29*, *44*), which are often used to search for singularities. The key innovation of our approach is that the initial condition is directly obtained from the CAV field and is therefore representative of turbulence.

**Fig. 4.**
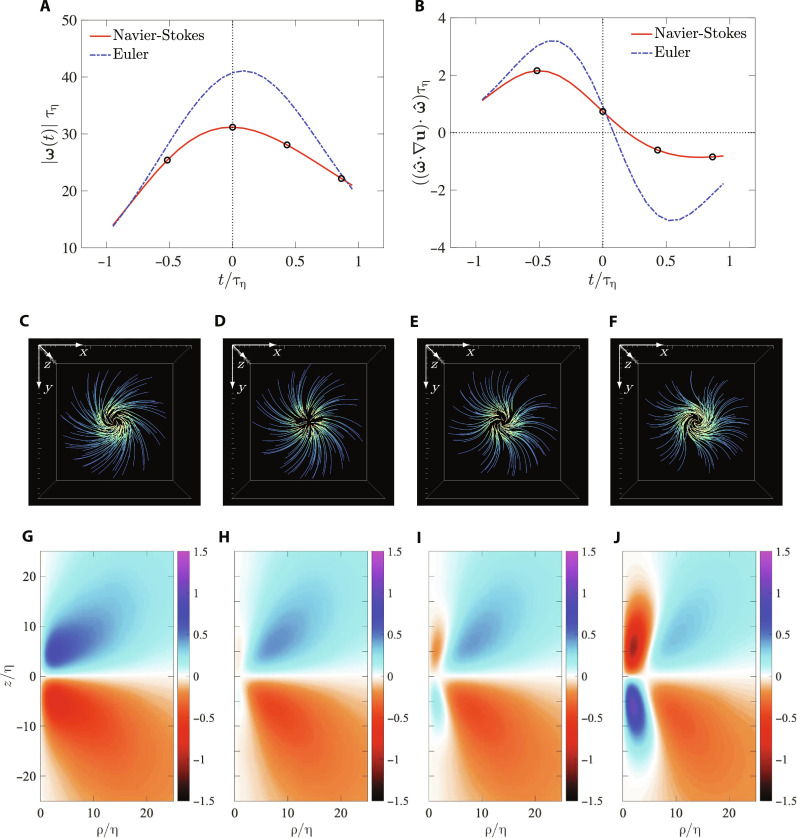
Temporal dynamics of an isolated vortex structure. (**A**) Evolution of vorticity magnitude at **r** = 0, as a function of nondimensional time *t*/τ_η_, where τ_η_ = 〈Ω〉^−1/2^ is the Kolmogorov timescale. The Euler case corresponds to the result when viscosity is set to zero. (**B**) Evolution of total stretching, also at **r** = 0. (**C** to **F**) The structure of vortex lines (top view) corresponding to the four time instants marked in (A) and (B). Initially, the vortex lines only have a positive twist, but later, an anti-twist develops in the center. (**G** to **J**) The twist component ${\bar{\omega }}_{\theta }$ also at the same four time instants, again showing the emergence of anti-twist.

The temporal evolution of the isolated vortex structure is shown in Fig. 4. Figure 4A shows the temporal evolution of the vorticity magnitude at the center of the core. The initial state is the same for both Navier-Stokes and Euler simulations, and the initial time is taken to be negative for convenience, with *t* = 0 corresponding to a later time when maximum amplitude is reached in the Navier-Stokes simulation. We first focus on the Navier-Stokes simulation and will come back to the Euler case. For *t* > 0, the vorticity magnitude starts decreasing. The evolution of the flow, via vortex lines, is shown in Fig. 4 (C to F), corresponding to the times marked in Fig. 4A. For convenience, the top view is shown [similar to Fig. 2 (D to F)]. It can be seen that, initially (field in Fig. 2C), the vortex lines all twist clockwise, when going outward from the origin. However, as time progresses, an anti-twist emanates from the vortex core (with lines going counterclockwise and essentially acting to oppose the regular clockwise twist in the background). To better quantify this, Figure 4 (G to J) shows the corresponding contour fields of the twisting component ${\bar{\omega }}_{\theta }$ for the same times. Once again, we notice (in Fig. 4G) that the twisting is positive everywhere at the initial time. However, in Fig. 4H, we can clearly see that as vorticity is self-amplifying, an anti-twist is also simultaneously produced. In Fig. 4 (I to J), this counter twist region grows bigger, corresponding to further attenuation of vorticity.

In Fig. 4B, the corresponding temporal evolution of the total stretching, acting on the vorticity at **r** = 0, is shown (as also defined in Eq. 5). We notice that, initially, the stretching increases with time, enabling vorticity amplification (as shown in Fig. 4A). However, even before maximum of vorticity magnitude is reached (at *t* = 0), the stretching starts to decrease, corresponding to the emergence of the anti-twist. As the anti-twist region grows, the net stretching eventually becomes negative.

As mentioned earlier, we also consider the Euler simulation, which corresponds to the Navier-Stokes case with viscosity set to zero. The behavior of the Euler simulation is shown in both Fig. 4 (A and B). Essentially, the same qualitative behavior is obtained as the Navier-Stokes case, with some minor quantitative differences. For instance, the maximum amplitude of vorticity is slightly higher for the Euler run and reached at *t* > 0, essentially because viscosity still plays a noticeable role in attenuating vorticity (which is expected to get smaller at very high Reynolds numbers, which are now inaccessible in DNS). Likewise, although not shown, the same qualitative behavior is obtained for the Euler case, as the Navier-Stokes case shown in Fig. 4 (C to J).

On the basis of the results shown in Fig. 2, the following picture emerges. The initial condition sets up for self-amplification of the isolated vortex under its own twist. However, as it undergoes amplification, the anti-twist emerges from the core, leading to attenuation of the vortex. Thus, it follows that this self-attenuation mechanism stems from nonlinear dynamics of the equations, implying that they already encode a nonlinear mechanism to regularize extreme events. While this conclusion can be drawn from the Navier-Stokes run itself, the Euler simulation further reinforces it. The presence of such a mechanism was recently hinted in (*23*). Our results here establish the anti-twist in the CAV as the key statistical feature in the vorticity field that enables this self-regularization.

## DISCUSSION

In this work, we have used both well-resolved numerical simulations and laboratory experiments to analyze the behavior of vortex lines in the neighborhood of intense vorticity regions, which are signatures of potential singularities of Navier-Stokes turbulence. We use the unique CAV framework (*30*, *31*), which allows us to change the reference frame and align the observer with vorticity undergoing amplification. In this frame, the nonlinear self-stretching of vortex lines, the only possible source of singularity, can be solely expressed in terms of the twist component of the CAV field. Since vorticity amplification is an intrinsic property of turbulence, a positive twist in the CAV field is expected and observed, also in accordance with previous works (*31*, *32*). However, we find that the CAV field remarkably generates a negative anti-twist which locally attenuates intense vorticity. By isolating a vortex structure which initially has only positive twist, we demonstrate the spontaneous emergence of the anti-twist when evolved under both Navier-Stokes and inviscid Euler dynamics. The development of the anti-twist, encoded in the nonlinearity, therefore provides an inviscid means to prevent singular behavior in Navier-Stokes equations (*12*).

The results established within the CAV framework in the current work relate to various recent observations in a similar context. In (*23*, *38*), a self-attenuation mechanism was identified in the nonlinearity of Navier-Stokes, by analyzing the nonlocal coupling between vorticity and strain fields. The emergence of the anti-twist in vortex lines to attenuate amplification essentially provides an explanation for the self-attenuation mechanism. In this regard, it also would be interesting to understand the results obtained here (using the CAV framework), in the more traditionally used eigenframe of strain, particularly by analyzing the dynamics of alignments between vorticity and eigenvectors of strain, together with the sign of the intermediate eigenvalue (*16*, *45*). Likewise, it could also be useful to relate the behavior of the CAV field with that of the pressure field (*46*, *47*).

It is also worth considering how the CAV framework is related to other statistical approaches to turbulence. The CAV field also naturally relates to the instanton framework for the vorticity in 3D turbulence, which aims at capturing the statistics of extreme events (*48*). Recent instanton calculations, analyzing extreme vorticity events in turbulence, also remarkably showed signs of an emergent anti-twist (*48*, *49*), motivating further work on the implications of this feature for extreme event statistics.

While the emergence of the self-regularizing anti-twist ensures smoothness of vortex lines, an important future task would be to leverage this physical mechanism to establish rigorous bounds on vorticity amplification (*13*, *27*). In this regard, earlier works on core dynamics of an isolated vortex column (*50*) bear noticeable similarity to our own analysis presented here. In (*50*), studying an isolated axisymmetric vortex column—albeit in an idealized and simplified setting compared to the CAV field—the authors identified a differential rotation mechanism arising due to variations in vortex core size, which naturally enables twisting of vortex lines. Such variations in core size are also obtained in vortex structures identified using the CAV field and, as discussed earlier, are notably absent in Burgers’ vortices. Together with the current results, this motivates further work toward a comprehensive understanding of vortex structures in turbulence, their potentially self-regularizing dynamics (especially in connection to the Navier-Stokes regularity problem), and ultimately toward establishing a statistical description of turbulence.

Last, it is worth emphasizing that although the current work focuses on incompressible fluid turbulence, vortex lines are prevalent in various other phenomena, such as quantum turbulence, plasmas, and electromagnetism. In this context, the unique CAV framework and insights derived from it offer a unique approach to analyzing these diverse and challenging problems.

## MATERIALS AND METHODS

### Direct numerical simulations

The numerical data are obtained through DNS of the INSE

$$\frac{\partial u}{\partial t}+u\cdot \nabla u=-\nabla P+\nu \nabla^{2}u+f$$ (6)

where **u** is the divergence-free velocity field (∇ · **u** = 0), *P* is the kinematic pressure, ν is the kinematic viscosity, and **f** corresponds to a large-scale forcing used to maintain a statistically stationary state (*51*). The simulations correspond to the canonical setup of homogeneous and isotropic turbulence, with periodic boundary conditions on a cubic domain, which is ideal for studying small scales and hence extreme events at highest Reynolds numbers possible (*19*). The domain length in each direction is *L*_0_ = 2π and discretized using *N* points with uniform grid spacing Δ*x* = *L*_0_/*N*. We solve the equations using a massively parallelized version of the well-known Fourier pseudo-spectral algorithm of Rogallo (*52*), and the resulting aliasing errors are controlled by a combination of grid shifting and spherical truncation (*53*). Whereas for time integration, we use explicit second-order Runge-Kutta, with the time step Δ*t* subject to the Courant number (*C*) constraint for numerical stability, i.e., Δ*t* = *C*Δ*x*/‖**u**‖_∞_ (where ‖·‖_∞_ is the *L*^∞^ norm).

The DNS database along with other simulation parameters is shown in Table 1 and is the same as used in numerous recent works focused on small-scale intermittency in turbulence, see, e.g., (*16*, *23*, *25*, *39*, *46*, *54*, *55*), which establish the reliability and veracity of the data. The Taylor-scale Reynolds number *R*_λ_ is in the range 140 to 1300. To precisely compare with experiments (described next), we have additionally performed a new simulation at *R*_λ_ = 200, which was not reported before. The small-scale resolution in pseudo-spectral DNS is given by the parameter *k*_max_η, where $k_{\text{max}}=\sqrt{2}N/3$ is the maximum resolved wave number on a *N*^3^ grid and η is the Kolmogorov length scale. Equivalently, one can use the ratio Δ*x*/η (≈2.96/*k*_max_η). For all of our runs, we have very high spatial resolution, going up to *k*_max_η ≈ 6, to appropriately resolve the extreme events (*20*, *38*). This resolution can be compared to the one used in comparable numerical investigations of turbulence at high Reynolds numbers, which are mostly in the range 1 ≤ *k*_max_η ≤ 1.5 (*19*, *56*)—which do not resolve the extreme events adequately (*20*).

**Table 1.** Simulation parameters for the DNS runs. The Taylor-scale Reynolds number (*R*_λ_), the number of grid points (*N*^3^), spatial resolution (*k*_max_η), ratio of large-eddy turnover time (*T*_E_) to Kolmogorov timescale (τ_K_), length of simulation (*T*_sim_) in statistically stationary state, and the number of instantaneous snapshots (*N*_s_) used for each run to obtain the statistics.

*R*_λ_	*N* ^3^	*k*_max_η	*T*_E_/τ_K_	*T*_sim_	*N*_s_
140	1024^3^	5.82	16.0	6.5*T*_E_	24
200	1024^3^	3.72	24.8	6.0*T*_E_	24
240	2048^3^	5.70	30.3	6.0*T*_E_	24
390	4096^3^	5.81	48.4	4.0*T*_E_	28
650	8192^3^	5.65	74.4	2.0*T*_E_	35
1300	12,288^3^	2.95	147.4	20τ_K_	18

### Experiments

The experimental data constitute 2 × 10^5^ statistically independent snapshots of the turbulent velocity field in a 1-cm^3^ measurement volume near the mean-field stagnation point of a von-Kármán swirling water flow, at *R*_λ_ ≈ 200 (*34*). The flow facility consists of a 48–cm–inner diameter, 58-cm-tall stainless steel cylinder with internal baffles and filled with deionized water maintained at 21.2° ± 0.5°C and agitated by two 25-cm-diameter counter-rotating impellers rotating at 0.2 Hz (*57*). Near the geometric center of this vessel, homogeneous and axisymmetric turbulence is generated with large-eddy length scale *L* ≡ *u*′^3^/ϵ ≈ 77 mm and Kolmogorov length scale η ≈ 210 μm. The 3D velocity field was measured using scanning PIV (*35*). Tracer particles (6-μm-diameter polymethyl-methacrylate microspheres with specific gravity of 1.22) were seeded into the flow at a density of approximately 1 particle per (1.4η)^3^ and illuminated by a 4.7η-thick laser sheet, which was rapidly scanned across the measurement volume at 250 Hz using a galvanometer mirror scanner. The tracers were observed in forward scatter orientation at ±45° to the laser sheet, imaged at a spatial resolution of 20 μm per pixel (1:2 optical magnification) by a pair of Phantom v640 high-speed cameras at 15 kHz. Each scan generated 54 of 512 × 512 pixel stereo image pairs, which were used to generate tomographic reconstructions of the tracer distribution. Reconstructions from sequential scans were cross-correlated using a multipass PIV algorithm, with a final interrogation window size of 3.2η, for an effective spatial resolution of around 1.6η and vector spacing 0.8η. This yielded 3D, three-component velocity field measurements on a regular grid over a (42η)^3^ measurement volume.

### Evaluation of nonlinear term from CAV

Taking the dot product of Eq. 1 with **ω**, the equation for enstrophy Ω = ∣**ω**∣^2^ is given as

$$\frac{1}{2}\frac{D\Omega }{\mathrm{Dt}}=\left(\omega \cdot \nabla u\right)\cdot \omega +\nu \omega \cdot \left(\nabla^{2}\omega \right)$$ (7)

The amplification of enstrophy is engendered by the nonlinear term (**ω** · ∇ **u**) · **ω**. The conditional expectation 〈(**ω** · ∇ **u**) · **ω**∣Ω〉 allows us to assess the strength of nonlinear amplification for a given enstrophy magnitude. It is more convenient to consider $\langle \left(\hat{\omega }\cdot \nabla u\right)\cdot \hat{\omega }|\Omega \rangle =\langle \left(\omega \cdot \nabla u\right)\cdot \omega |\Omega \rangle /\Omega$, since it provides the effective stretching, irrespective of the strength of the vorticity (*16*, *45*). As mentioned earlier, the amplification term can be solely obtained in terms of vorticity itself, since the velocity field can be obtained from the vorticity field using Eq. 2. Combined with conditional averaging, the effective stretching can be related to the CAV field introduced in Eq. 4. To this end, we rewrite Eq. 2 using the index notation

$$u_{i}\left(x\right)=\frac{1}{4\pi }\int ‍\varepsilon_{\mathrm{ikl}}\;\omega_{l}\left(x+r\right)\;\frac{r_{k}}{r^{3}}\;dr$$ (8)

where *r* = ∣**r**∣. To evaluate the nonlinear term, we have to first obtain the gradient of **u**. By taking the derivative of Eq. 8 w.r.t. *x_j_* and using the theory of singular integrals (*58*), it can be shown that

$$\begin{array}{cc} \frac{\partial u_{i}}{\partial x_{j}}\left(x\right) & =-\frac{1}{2}\varepsilon_{\mathrm{ijk}}\omega_{k}\left(x\right)+\\ & \frac{1}{4\pi }\int ‍\varepsilon_{\mathrm{ikl}}\;\omega_{l}\left(x+r\right)\;\left[-\frac{\delta_{\mathrm{jk}}}{r^{3}}+3\frac{r_{j}r_{k}}{r^{5}}\right]\;dr \end{array}$$ (9)

where the integral on the right-hand side is taken in the sense of the principal value.

The nonlinear term now can obtained as

$$\begin{array}{c} {\hat{\omega }}_{i}{\hat{\omega }}_{j}\frac{\partial u_{i}}{\partial x_{j}}\left(x\right)=\frac{1}{4\pi }\int ‍\varepsilon_{\mathrm{ikl}}\;\omega_{l}\left(x+r\right){\hat{\omega }}_{i}\left(x\right){\hat{\omega }}_{j}\left(x\right)\\ \left[-\frac{\delta_{\mathrm{jk}}}{r^{3}}+3\frac{r_{j}r_{k}}{r^{5}}\right]\;dr \end{array}$$ (10)

Using ε*_ikl_*ω*_i_*ω*_j_*δ*_jk_* = 0 and with minor rearrangement, the expression then becomes

$$\begin{array}{c} {\hat{\omega }}_{i}{\hat{\omega }}_{j}\frac{\partial u_{i}}{\partial x_{j}}\left(x\right)=\frac{3}{4\pi }\int {\hat{\omega }}_{i}\left(x\right)\;\varepsilon_{\mathrm{ikl}}\frac{r_{k}}{r}\omega_{l}\left(x+r\right)\\ \frac{r_{j}}{r}{\hat{\omega }}_{j}\left(x\right)\;\frac{1}{r^{3}}\;dr \end{array}$$ (11)

Reverting back to vector form, we can write

$$\begin{array}{c} \left(\hat{\omega }\cdot \nabla u\right)\cdot \hat{\omega }=\frac{3}{4\pi }\int \hat{\omega }\left(x\right)\cdot \left[\hat{r}\times \omega \left(x+r\right)\right]\\ \frac{\hat{r}\cdot \hat{\omega }\left(x\right)}{r^{3}}\;dr \end{array}$$ (12)

Without loss of generality, we can apply the conditioning for the state **ζ** = **ω**(**x**)

$$\begin{array}{c} \langle \left(\hat{\omega }\cdot \nabla u\right)\cdot \hat{\omega }|\zeta \rangle =\frac{3}{4\pi }\int \hat{\zeta }\cdot \left[\hat{r}\times \langle \omega \left(x+r\right)|\zeta \rangle \right]\\ \frac{\hat{r}\cdot \hat{\zeta }}{r^{3}}\;dr \end{array}$$ (13)

Using 〈**ω**(**x** + **r**)∣**ζ**〉 from Eq. 4, together with $\hat{\zeta }=\hat{\omega }\left(x\right)=e_{z}$ and $\hat{r}=\gamma e_{z}+{\left(1-\gamma^{2}\right)}^{1/2}e_{\rho }$ , it can be readily shown that

$$\hat{\zeta }\cdot \left(\hat{r}\times \langle \omega \left(x+r\right)|\zeta \rangle \right)={\bar{\omega }}_{\theta }{\left(1-\gamma^{2}\right)}^{1/2}$$ (14)

whereas the term $\hat{r}\cdot \zeta =\hat{r}\cdot e_{z}=\gamma$, leading to the result

$$\langle (\hat{\omega }\cdot \nabla u)\cdot \hat{\omega }|\zeta \rangle =\frac{3}{4\pi }\int ‍\frac{\gamma {\left(1-\gamma^{2}\right)}^{1/2}\;{\bar{\omega }}_{\theta }}{r^{3}}\;dr$$ (15)

Last, a simple transformation to cylindrical coordinates, with ρ = *r*(1 − γ^2^)^1/2^, *z* = *r*γ, and *d***r** = ρ*d*ρ*d*θ*dz*, with integration limits ρ ∈ [0, ∞], θ ∈ [0, 2π], and *z* ∈ [−∞, ∞] gives the result shown in Eq. 5. Since ω_θ_ does not depend on θ and is an odd function in *z*, we have integrated out *d*θ and appropriately changed the integration limits to *z* ∈ [0, ∞]. Note that the scalar Eq. 15 is a function of the vorticity magnitude only. Therefore, we can equivalently choose the enstrophy as the conditioning variable in Eq. 5.

### Numerical evaluation of the CAV field

The CAV field defined in Eq. 4 can be extracted from experimental and DNS data using two distinct methods. We note that we have used both these methods, and the results are essentially identical. Nevertheless, both the methods have certain advantages and disadvantages, so it is worth discussing them both.

#### Binning and sampling approach

The first method entails straightforward conditional averaging by simply accruing samples of vorticity vectors at any two spatial locations. For instance, consider two spatial locations **x**_1_ = **x** and **x**_2_ = **x** + **r** at time *t*, and the corresponding vorticity vectors **ω**_1_ = **ω**(**x**_1_, *t*) and **ω**_2_ = **ω**(**x**_2_, *t*); our task is to essentially evaluate 〈**ω**_2_∣**ζ** = **ω**_1_〉 for all combinations of the conditioning variable **ζ** = **ω**_1_ and the separation vector **r**. As noted earlier, because of statistical isotropy, the CAV field only depends on three scalar variables, viz. Ω = ∣**ζ**∣^2^, *r* = ∣**r**∣, and $\gamma =\hat{r}\cdot \hat{\zeta }$ , which can be readily calculated using **ω**_1_ and **r**. We can also obtain the orthogonal basis vectors (**e***_z_*, **e**_ρ_, and **e**_θ_) as defined immediately after Eq. 4. Thereafter, the components ${\bar{\omega }}_{z},{\bar{\omega }}_{\rho },\text{and}\;{\bar{\omega }}_{\theta }$ are easily computed by taking simple dot products of these unit vectors with the **ω**_2_ vector, i.e., ${\bar{\omega }}_{z,\rho ,\theta }=e_{z,\rho ,\theta }\cdot \omega_{2}$ . Thus, for each sample pair, we can obtain the three scalar variables Ω, *r*, and γ and also the three components ${\bar{\omega }}_{z,\rho ,\theta }$ . The scalar variables can be sampled into discrete bins, and the components can be appropriately averaged over all the samples to obtain the conditional expectations $\langle {\bar{\omega }}_{z,\rho ,\theta }|\Omega ,r,\gamma \rangle$ . The CAV field itself can then be readily reconstructed from these components for any choice of conditioning vorticity and separation vector as necessary.

Evidently, the above method is simple and straightforward in application. By taking two spatial points at a time and scanning through all such samples, the conditional averages can be obtained very easily. However, for a *N*^3^ grid, the total number of pairs that can be formed scales as 𝒪(*N*^6^), which is obviously prohibitively expensive. Thus, only a limited number of pairs can be counted using this method. In addition, complexities arise in data processing, since the domain is distributed across multiple processors, see, e.g., (*56*, *59*), for an exposition on similar issues arising in the context of processing pairs of particle trajectories. Thus, for convenience, we only process pairs of samples along given Cartesian grid lines. Even then, the computational cost can be very high, since for *N* points along a grid line, there are 𝒪(*N*^2^) samples, implying a total of 𝒪(*N*^4^) samples for the entire grid. Since we are primarily concerned with the localized structure around intense vorticity, we restrict the samples to typically *r* ≲ 100η (which still amounts to about 100 to 1000*N*^3^ samples).

#### Spectral approach

The difficulty of the first approach can be mitigated by using a spectral approach to directly evaluate the entire CAV field. To that end, we can rewrite the conditional expectation in Eq. 3 as

$$\langle \omega \left(x+r,t\right)|\zeta \rangle =\frac{1}{f\left(\zeta \right)}\langle \omega \left(x+r,t\right)f\prime \left(\zeta ;x,t\right)\rangle$$ (16)

where *f*′(**ζ**; **x**, *t*) = δ[**ω**(**x**, *t*) − **ζ**] is the fine-grained probability density function (PDF) of vorticity, and *f*(**ζ**) = 〈*f*′(**ζ**)〉 is simply the PDF of vorticity. For details pertaining to the above relation, see, e.g., Appendix H in (*60*). Thus, the CAV field can be obtained as a convolution between the vorticity field and the fine-grained PDF. This convolution can be evaluated spectrally for any chosen conditioning vorticity as follows. First, we define the indicator function *I*(**x**) = δ[**ω**(**x**, *t*) − **ζ**], which is set to unity at every grid point where the vorticity condition is satisfied and zero everywhere else. Note that to satisfy the condition, both the vorticity magnitude and orientation must be matched. For the magnitude, we simply consider the same finite bins for enstrophy as used before (in the binning approach), whereas for the orientation, we consider vorticity aligned with any Cartesian grid direction, within a small tolerance (of about 5%). Thereafter, a Fourier transform of the vorticity field and the indicator function is performed. The Fourier transform of the CAV field can be defined from Eq. 16 as

$$\tilde{\bar{\omega }}\left(k,\zeta \right)=\frac{1}{f\left(\zeta \right)}\langle {\tilde{\omega }}^{*}(k)\;\tilde{I}\left(k\right)\rangle$$ (17)

where $\left(\tilde{\cdot }\right)$ denotes the Fourier coefficient, and **k** is the wave vector. Thus, the CAV field can be obtained in spectral space by performing a convolution of the vorticity and indicator function and lastly in physical space by taking an inverse transform.

Evidently, the main advantage of the spectral approach is that it provides a direct evaluation of the CAV field in the entire domain at the same cost as a 3D fast Fourier transform, which is *N*^3^log_2_*N*, which is obviously more cost-effective than the earlier binning approach. However, it also only provides the CAV field for one chosen conditional magnitude at a time, i.e., the entire procedure has to be repeated for every conditional magnitude desired. Since the number of chosen conditional values is fixed and does not depend on *N*, the overall cost for this approach still scales as *N*^3^log_2_*N*. As mentioned earlier, we have used both the approaches, and they essentially give identical results (as expected). However, the spectral approach is particularly useful in setting up the initial condition in Fig. 4, since it needs to be performed only for one chosen value of conditional enstrophy and also for the entire domain.

### Initial condition for dynamical evolution

The vortex structure which serves as the initial condition in Fig. 4 can be extracted in two ways. For the first approach, the CAV field is extracted at an earlier time, at which point the conditional vorticity is weak and still undergoing amplification. This can be done by generalizing the CAV framework to two times, i.e., 〈**ω**(**x** + **r**, *t*_1_)∣**ω**(**x**, *t*_0_)〉, where *t*_1_ < *t*_0_, and *t*_0_ corresponds to the time at which maximum enstrophy is reached. In this approach, one requires Lagrangian trajectories stored with the Eulerian data in DNS, which can be expensive.

The second approach is to use the CAV field as obtained earlier and briefly evolve it backward in time until an initial condition is obtained where the vorticity is relatively weak and only the regular twisting of vortex lines exists. This approach is far more efficient for implementation, and hence, we use this here. However, we note that we have also verified that using the first approach and both of them essentially give the same outcome (with some minor quantitative differences).
