# Supplementary material for: Twisting vortex lines regularize Navier-Stokes turbulence
Source: Sci Adv. 2024 Sep 13;10(37):eado1969. doi: 10.1126/sciadv.ado1969 (PMC11421575; doi:10.1126/sciadv.ado1969)
Supplement: Supplementary file 1 — Figs. S1 to S3 [file sciadv.ado1969_sm.pdf]

Science Advances
AAAS

Supplementary Materials for

Twisting vortex lines regularize Navier-Stokes turbulence

Dhawal Buaria *et al.*

Corresponding author: Dhawal Buaria, dhawal.buaria@ttu.edu

Sci. Adv. **10**, eado1969 (2024)
DOI: 10.1126/sciadv.ado1969

This PDF file includes:

Figs. S1 to S3

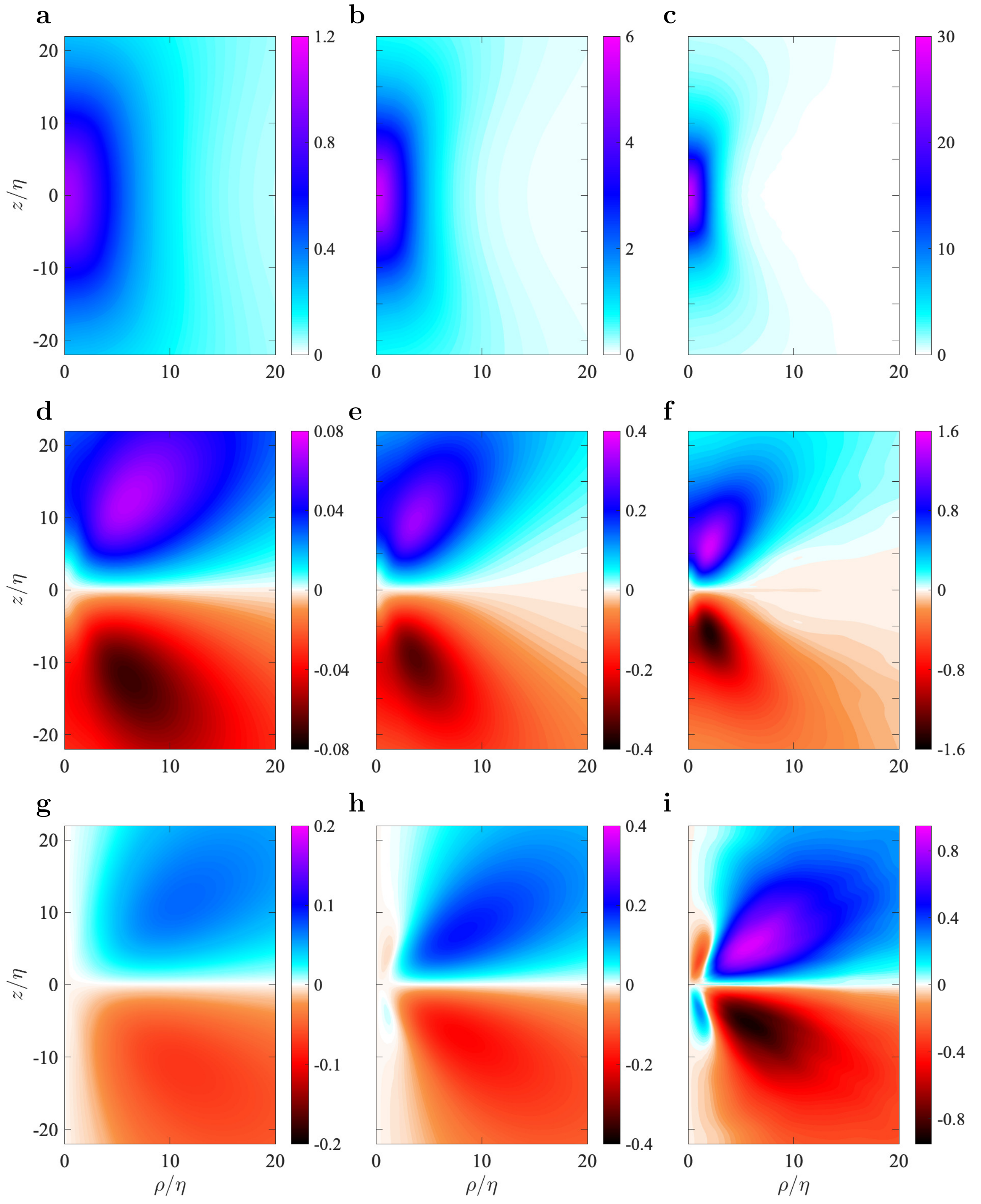

SUPPLEMENTARY FIG. 1. **Components of the CAV field**. Extending Fig. 2 of the main text, this figure shows all the components of the CAV field. a-c) The axial component ω_z at $R_\lambda = 1300$, for conditioning magnitudes $\Omega/\langle\Omega\rangle = 1, 30, 1000$, respectively. d-f) The radial component ω_r for same conditioning magnitudes. g-i) the azimuthal component ω_θ (identical to Fig. 2g-i of the main text).

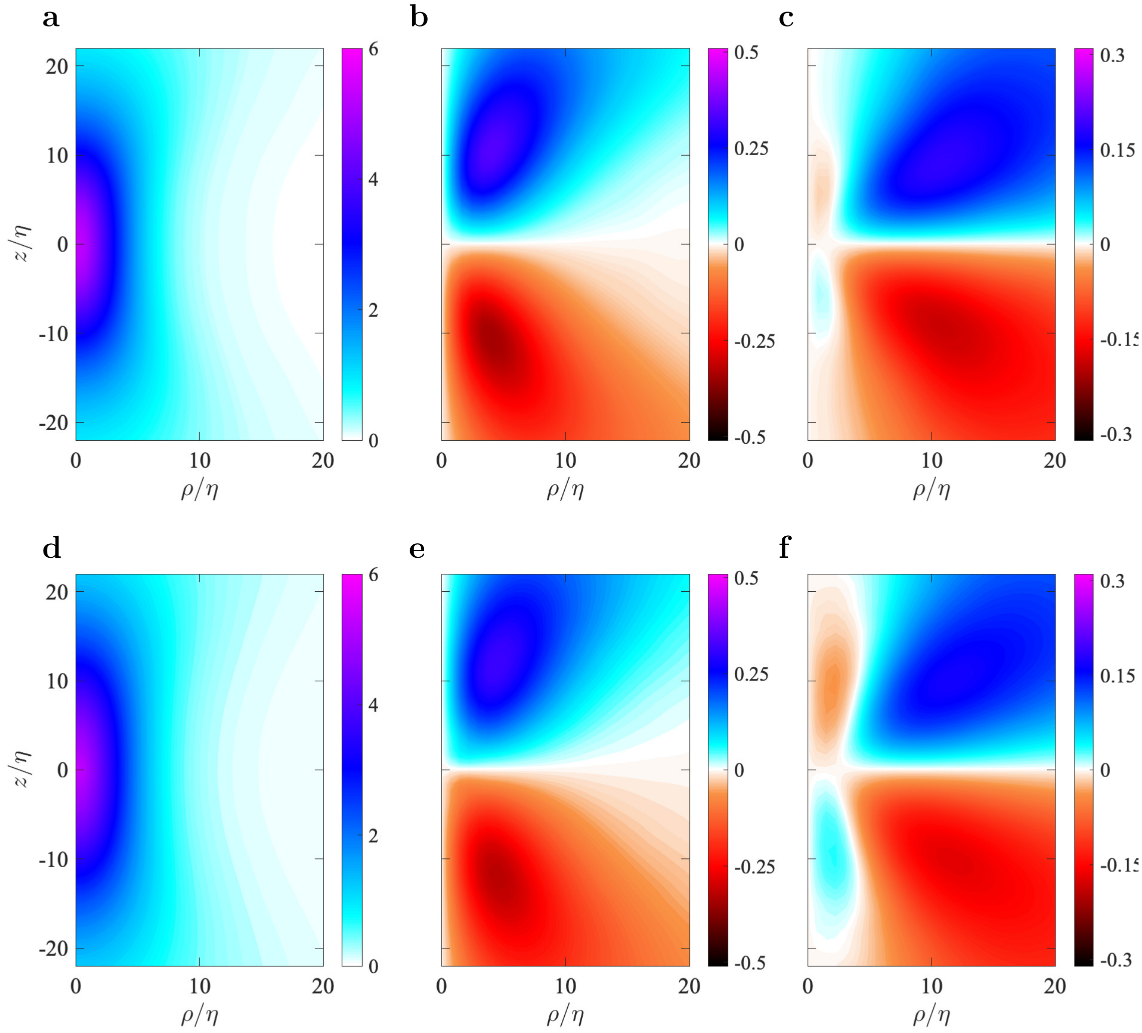

SUPPLEMENTARY FIG. 2. **Comparing CAV fields from DNS and experiments**. Extending Fig. 3 of the main text, this figure shows all the components of CAV field from DNS and experiments at $R_\lambda = 200$ and conditioning magnitude $\Omega/\langle\Omega\rangle = 30$. a-c) The components ω_z, ω_r and ω_θ, respectively from DNS. d-f) The same components from experiments. Panels c and f are respectively identical to Fig. 3c-d of the main text.

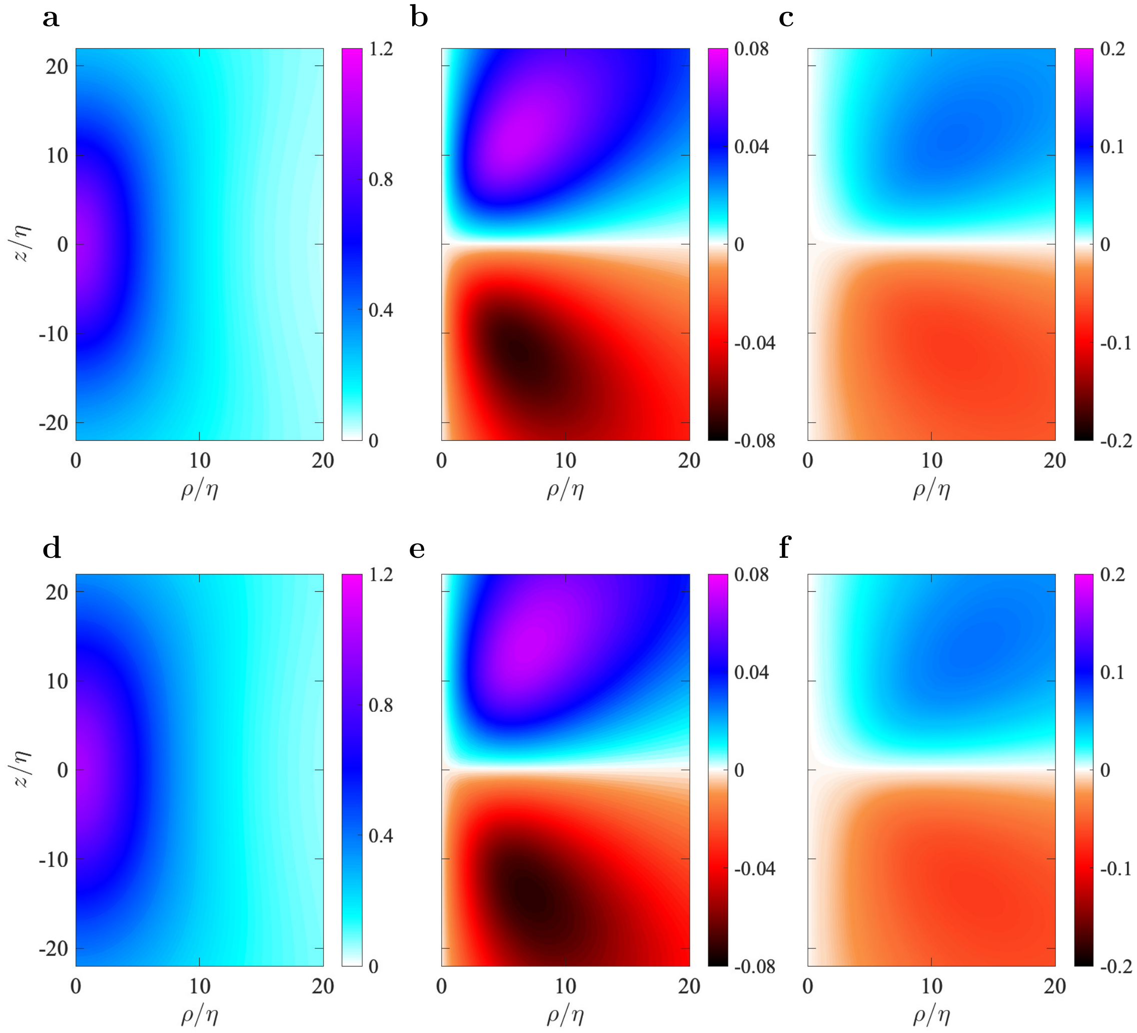

SUPPLEMENTARY FIG. 3. **Comparing CAV fields from DNS and experiments**. Same as Supplementary Fig. 3, but for conditioning magnitude $\Omega/\langle\Omega\rangle = 1$. There is no anti-twist in the CAV field for the mean field (as also observed in Fig. 2 of the main text). Supplementary Figs. 2-3 show execellent correspondence between DNS and experiments.
